# An examination of the medicalization and pharmaceuticalization processes of anxiety and depressive disorders in Belgium between 2004 and 2013: how may both disorders be intertwined?

**DOI:** 10.1186/s13690-022-00943-x

**Published:** 2022-08-15

**Authors:** Van Looy Kilian, Van de Velde Sarah

**Affiliations:** Centre for Population, Family and Health, Department of Sociology, University of Antwerp, St-Jacobstraat 2-4, 2000 Antwerp, Belgium

**Keywords:** Medicalization of mental health, Depressive disorders, Anxiety disorders, Psychopharmacology, Medical sociology, Pharmaceuticalization

## Abstract

**Background:**

While medicalization and pharmaceuticalization trends of feelings of anxiety and depression have been described in great detail, an empirical examination of these trends is to date lacking. The current study fills this gap in the literature by mapping the use of psychotropic medicines for feelings of anxiety and depression between 2004 and 2013 in Belgium, as well as by examining whether a social gradient might act as a mediator.

**Methods:**

We analyzed data from three repeated cross-sectional waves (2004, 2008, and 2013) of the Belgian National Health Interview Survey (HIS). Multinomial logistic regression was applied to estimate odds in psychotropic drugs use over the observed period.

**Results:**

Using an ideal-typical distinction between traditional anxiety drugs (psycholeptics) and depression drugs (psychoanaleptics), we found that treatment methods for feelings of anxiety and depression were converging. Persons having feelings of anxiety consumed less psycholeptic drugs, in favor of psychoanaleptic drugs throughout the observed period. Moreover, these results were partially mediated by educational level. Persons with higher education were less likely to consume psychotropic drugs than those with lower education, suggesting a trend of demedicalization for feelings of anxiety and depression.

**Limitations:**

Our study observes a limited period, makes use of an ideal typical distinction between psycholeptic and psychoanaleptic drugs, and measurements may be biased by response-bias due to psychotropic drugs use.

**Conclusion:**

Our study shows that psycholeptics increasingly give way to psychoanaleptics in the treatment of both anxiety and depression, despite several scientists calling their effectiveness for both disorders into question.

**Supplementary Information:**

The online version contains supplementary material available at 10.1186/s13690-022-00943-x.

## Background

Anxiety and depression are the most prevalent mental disorders worldwide [1]. Approximately one in five persons experience at least one of both disorders during their lifetime, and these numbers continuously increase over time [2, 3]. In Belgium, around 10% of the population suffers from either (or both) AD or DD in 2018, a number that remained largely stable since 2013, but nearly doubled since 2004 [4]. We focus on the Belgian context, whereas its elaborate managed care system, in combination with tight (European) regulations concerning the consumption and prescription of medicines offers an interesting case.

There is ample research that focuses on either anxiety disorder (AD) or depressive disorder (DD), yet social scientists rarely examine their interrelatedness empirically [5, 6]. Only, to our knowledge, no empirical research exists that scrutinizes how processes of medicalization and pharmaceuticalization affected changes in treatment methods over time and how this differs between persons with feelings of anxiety versus feelings of depression. Our research aims to address this gap by investigating how the medicalization and consequent pharmaceuticalization of feelings of anxiety and depression evolved between 2004 and 2013, specifically focusing on psychotropic drugs consumption within the Belgian context.

In addition, we assess whether a social gradient might be a mediating factor. This second aim is guided by the great body of literature that deals with inequalities in both the access to and use of mental healthcare [7]. Our study will use education level as a proxy for this social gradient, as education is identified as one of the most fundamental causes of social disparities in healthcare use [8], with those who are higher educated generally taking on a more active role in the treatment process [9].

### Medicalization of feelings of anxiety and depression

In the literature, AD is generally defined as having an excessive reaction to a future threat [10]. In turn, DD is defined as having an excessive manifestation of sadness [11]. Nevertheless, “excessiveness” is not defined at all, leaving AD or DD diagnoses subject to individual interpretation, often in combination with diagnostic tools, such as symptom checklists [12]. These definitions and symptoms have indeed changed throughout the last decades [5, 13], with striking shifts that align with the first and second generations of medicalization [14]. This has had an undeniable impact on the way these disorders are perceived (by society, clinicians, or patients), and is directly linked to their diagnoses, prevalence, and treatment [12].

In sociology, the similarity between AD and DD is often emphasized, referring to their shared risk factors and social outcomes [3, 15]. Both disorders are sometimes seen as different sides of the same coin. Social constructionists even claim that distinctions between mental disorders are a consequence of medicalization, which is defined as the process by which a non-medical problem, behavior, or human condition is defined and/or treated as a medical problem [14], or as Horwitz classically describes: “transforming normality into pathology” [16]. Yet, the way mental disorders like AD and DD are medicalized, changed drastically throughout the years, resulting in contrasting diagnoses and treatment methods [14]. Consequently, this has led to very different outcomes for the numerous individuals that have been treated with either (or both) disorders and how society perceives these disorders and treatment methods [5, 17, 18].

A quintessential example of this are the shifts in the definition of AD and DD by the American Psychological Association (APA), which had (and still has) an eminent influence on the medicalization of feelings of anxiety and depression in most high-income countries, including Belgium. Over the last decades, the APA systematically widened its sphere of influence by monopolizing mental disorders, deciding which feelings should be classified as disorders and which should not [16, 19]. During the last century, the APA systematically increased the number of ADs, framing a growing number of personal characteristics as AD pathologies (e.g., shyness becoming social phobia) [20]. In turn, while DD was still quite obscure before the 1980s (with only a few persons qualifying for its severe symptoms and diagnostic criteria), this changed with the publication of the DSM-III: DD were increasingly becoming more generalized under the umbrella term of ‘major depressive disorder’ (MDD). For instance, the DSM-V discarded the contested ‘bereavement clause’, which excluded “normal” feelings of sadness from depression, induced by, for example, grieving a close death, thereby inevitably causing diagnoses to rise significantly [21]. In succession to AD, DD consequently became “psychiatry’s most marketable diagnosis” [22].

### Pharmaceuticalization of feelings of anxiety and depression in a managed care system

Traditionally, feelings of anxiety have been treated with tranquilizing psychotropic drugs, such as benzodiazepines, which could broadly be classified under the term *psycholeptics* [23]. On the other hand, feelings of depression are generally treated with stimulants such as selective serotonin reuptake inhibitors (SSRIs), classified as *psychoanaleptics* [24]. Now, however, treatment for both disorders seems to be converging [25]. In the current paper, we use this distinction as an ideal-typical dichotomy, allowing us to gauge the foundations of the medicalization processes of both disorders, through their pharmaceuticalization.

As medicalization continuously pushed the boundary on what should be deemed as sickness opposed to normality, pharmaceuticalization describes the process as how these persons should then be treated. The ‘rational use of medicines’ paradigm, which poses that individuals rationally choose which medicines they consume, became increasingly contested throughout the past two decades, “with diverse actors, social systems, and institutions [now] determining who uses what medications, how, when and why” [26].

We argue the aforementioned shifts in the delineation and definition of AD and DD went hand in hand with shifts in the prescription and consumption of psychotropic drugs. Stimulated by the rapid development of these drugs during the last century, the dominant idea grew to cure mental disorders with medications. Importantly, however, is these new treatment methods often merely suppressed symptoms, rather than eliminating their cause [13, 19]. Even so, these methods were continuously promoted by professional organizations and the pharma industry, first to clinicians, and then to the public, giving rise to the age of psychotropics [22].

However, while developments in psychoanaleptic drugs treatment initially showed promise, psycholeptics were increasingly perceived dangerous due to their addictive properties [25, 27]. As a result, in recent years, psychoanaleptics have become the go-to remedy for both feelings of anxiety and depression, and an increasingly wider array of other mental disorders as well [25, 27]. Aggressively pushed forward by the APA, the use of psychoanaleptics has increased steadily for all ages, genders, and ethnic-racial groups [25, 27]. There is an increasing overlap in how both AD and DD are treated, causing the conceptual lines between them to blur. Or, as Ehrenberg [28] notes: “Everything becomes depression, because antidepressants act on everything”.

In Belgium, mental healthcare policy largely follows the APA’s DSM recommendations for diagnoses and treatment options (see e.g. [29]). As such, it is estimated that nearly one-tenth of the adult Belgian population used an antidepressant in the past 30 days [4]. In turn, while still having a higher consumption rate in Belgium [4], the use of psycholeptics decreased or at least stabilized, especially for long-term use [30].

The aforementioned shifts in the medicalization and pharmaceuticalization of feelings of anxiety and depression occurred in an era of the emergence of managed care systems. Elaborate insurance schemes typically characterize these systems, mixing both basic (public) plans with more privatized “extra” plans for those who can afford it [31]. At best, basic security is offered to those most in need (as is the case in Belgium, see e.g. [32]), yet in more privatized national healthcare systems, such as the USA, this is less evident [33]. Industrialized healthcare systems are, however, hypothesized to converge to one another, i.e. leading privatized systems to become more centralized and vice versa (e.g. [34]). The same is true for Belgium. What types of treatment are refundable is constantly evaluated by government subsidiaries, such as the National Institute for Health and Disability Insurance (NIHDI). For example, in 2013, the Belgian Psychotropics Experts Platform (BelPEP) was founded as a result of a worrisome publication concerning the (over) use of psychotropic drugs within the Belgian population [35]. BelPEP [35] advised the NIHDI to restrict the use of these kinds of psychotropic drugs, particularly psycholeptics. This led to the formal Royal Resolution of September 6, 2017, significantly tightening prescription regulation, e.g., to individuals with a history of addiction.

### The social gradient within medicalization and pharmaceuticalization processes

Added to the already present social gradient within the prevalence of disorders such as AD and DD, inequalities also exist in the medicalization of both disorders, highlighting its complexity and diversity (e.g. [36]). During the first generation of medicalization, these inequalities were largely (re) produced by clinicians, for instance, by choosing who they ultimately prescribe certain medications or treatments to [37]. Moreover, it is argued that at least some prevalence disparities, such as individuals with a more precarious socioeconomic position being more likely to have feelings of anxiety and depression (see e.g. [38]), are partially mediated by this process, whereas individuals with higher SES might simply enjoy better healthcare on average [7, 39].

The nature of this stratification changed during the second generation of medicalization. With healthcare becoming more an individual responsibility, patients became more active in their personal healthcare management. In the previous century, treatment with psycholeptic drugs was highly promoted to the middle and upper classes, leaving the lower classes to miss out [5, 14]. It should however be noted that, in Belgium, marketing of such medications and treatments had already been more strictly regulated than in e.g., the USA. Nonetheless, when the perception towards these medications shifted and the upper class abandoned them, they only just started to become available for the lower classes, leaving them to become their new primary users [5]. This is in line with more recent research on pharmaceuticalization, which states that sole processes of biomedicalization are insufficient to explain for shifts in medicine use, rather are they exacerbated by other drivers, such as, indeed, consumption patterns (i.e., for different social groups) [40].

Educational level seems of particular importance in this context. While persons with lower educational level are generally more at risk of feelings of anxiety [41] and feelings of depression [42], it also influences health care behavior in patients. Persons with a higher educational level are generally less likely to consume psychotropic drugs [43] and take on a more active role as a patient [9, 14]. Furthermore, they tend to be more informed concerning different treatment options, which helps them gain access to newer forms of treatment [9]. Persons with lower education are, however, more likely to consume psycholeptics such as benzodiazepines [43]. Additionally, persons with higher education increasingly opt for alternative medicine, with a great emphasis on preventative health behavior [44]. This suggests there is an ongoing trend of demedicalization and shifts in help-seeking behavior, especially in those that are higher educated [9, 44].

### Aims and hypotheses

The aim of this study is to describe how the medicalization of feelings of anxiety and depression in Belgium may have shifted throughout the observed period. That is, we describe a “shift” as when the odds of the general use of psychoanaleptic medicines becomes greater than psycholeptic medicines (or vice versa) at a certain point in time, when compared to other points in time. We do not claim to describe longitudinal trends with our cross-sectional data. Based on the literature, we expect that a shift from psycholeptic to psychoanaleptic drugs consumption will have occurred (hypothesis 1), and that this shift was most likely and outspoken for a group of persons having more feelings of anxiety with them being less likely to consume psycholeptic drugs, in favor of psychoanaleptic drugs (hypothesis 2). Lastly, we expect that a higher educational level corresponds with a greater likelihood to consume either type of psychotropic drugs compared to those with lower education (hypothesis 3). This would fit in with current demedicalization trends, where we expect a general downward trend in either type of psychotropic drugs consumption to be highest in those with higher educational levels (hypothesis 3b).

## Methods

### Data

This study used data from the third (2004), fourth (2008), and fifth (2013) waves of the Belgian Health Interview Survey (HIS), executed by Sciensano, and commissioned by the Belgian Federal Government [45]. This data was accessible upon request to the Privacy Commission. Using nationally representative samples via a stratified, multistage, clustered design, the HIS administered standardized questionnaires through face-to-face interviews, both written and orally, on the household level and the individual level. From each household, up to four persons were selected for the individual interview, though this study selected only the primary individual to provide for independent measures, as is required for regression analyses. Moreover, we only included persons aged between 18 and 75 in our analyses. While the HIS was also executed in 1998, 2001, and 2018, we excluded these waves since they do not provide consistent measures for our used variables. The final sample comprised of 7214 respondents.

### Variables

#### Type of psychotropic drugs used

Making use of the Anatomical Therapeutic Chemical classification (ATC), which is recommended by the World Health Organization [46] as an international standard for drug utilization studies, respondents’ psychotropic drugs use was assessed. This resulted in a categorical variable, measuring specifically the use of psycholeptic (ATC code N05) and psychoanaleptic (ATC code N06) medication. The variable consisted of four categories: use of (1) neither medications, (2) only psycholeptic, (3) only psychoanaleptic drugs, or (4) use of both medications simultaneously.

#### Wave

The three waves of data collection (2004, 2008, and 2013) were used as indicators of time-periods. Since 2008 is the midpoint of our measures, we opted to take this wave as the reference. In doing so, this allowed us to better interpret and discuss our regression results.

*Feelings of anxiety* were measured through a ten-item scale within the Symptom Checklist Revised (SCL-90-R) scale [47]. Respondents were asked to indicate how often they have certain feelings or expressed specific behaviors (feeling fearful, heart pounding, nervousness, trembling, suddenly scared for no reason, feeling tense, spells of terror or panic, feeling so restless you could not sit still, feeling something bad is going to happen or thoughts and images of a frightening nature). Response categories ranged between 1 (not at all) and 5 (extremely). The scale total was the sum of all item-responses, divided by ten, and was reported as the mean score for feelings of anxiety, with a maximum of 5. This variable thus measured the intensity of feelings of anxiety. A reliability analysis shows a Cronbach’s Alpha of 0.90 for the anxiety scale.

*Feelings of depression* were measured via a 13-item scale within the SCL-90-R. Respondents were asked to indicate how often they had certain feeling or expressed specific behaviors (worry too much about things, feeling no interest in things, loss of sexual interest or pleasure, thoughts of ending your life, crying easily, feelings of being trapped or caught, blaming yourself for things, feeling lonely, feeling blue, feelings of hopelessness about the future, feelings everything is an effort and feelings of worthlessness). Response categories ranged between 1 (not at all) and 5 (extremely). The scale total was the sum of all item-responses, divided by 13 and was reported as the mean score for feelings of depression, with a maximum of 5. This variable thus measured the intensity of feelings of depression. A reliability analysis shows a Cronbach’s Alpha of 0.92 for the depression scale.

*Level of education* was measured through the highest obtained educational degree of the respondent and distinguished between (1) no diploma, (2) secondary education degree, and (3) higher education degree. Finally, a categorical *help-seeking behavior* variable was included, assessing having either visited a general practitioner (GP) and/or a psychologist in the past 12 months. It consisted of four categories: having visited neither (1), having visited only a GP (2), and having visited only a psychologist (3), and having visited both a psychologist and GP [3].

#### Control variables

Our analyses were controlled for *gender* and *age*. We also controlled for the curvilinear association between age and our outcome variables (see e.g., the work of Mirowsky and Ross, [48]).

### Statistical procedures

**Fig. 1:**
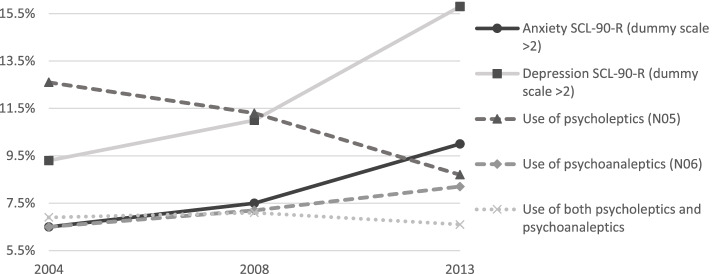
Anxiety, depression, and medicine use as a proportion of the population, in 2004, 2008, and 2013. Own calculations via the Belgian Health Interview Survey

**Fig. 2:**
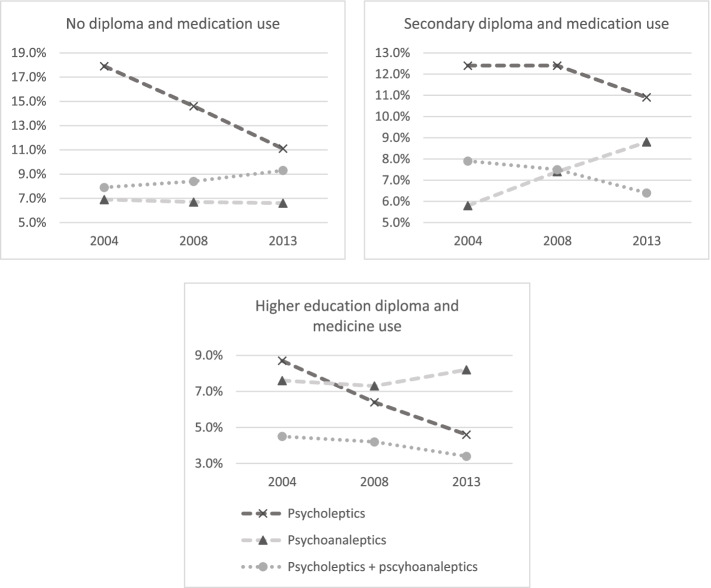
Medicine use as a proportion of educational level subpopulations, in 2004, 2008, and 2013. Own calculations via the Belgian Health Interview Survey

In a first step, we present a visualization of time trends for psycholeptics and psychoanaleptics consumption as a proportion of the population, compared to having AD and DD feelings, which are presented in Fig. 1. Figure 2 presents the association between educational level and psycholeptic and psychoanaleptic drugs consumption as a proportion, throughout the observed period. Note that, for both Figs. 1 and 2, we make use of cross-sectional data. The lines in the graph thus visualize the measures of three moments in time, namely 2004, 2008, and 2013. We make use of line graphs, whereas this offers the best visualization of the interrelatedness of all variables involved. Descriptives of all included variables are presented in the Appendix Table A1.

In a next step, our hypotheses were tested using multinomial logistic regression analyses. Models 1, 2, and 3 are presented in Table 1. In model 1, we analyzed the effect of wave on the psychotropic drugs use outcomes, establishing the effect of time trends, which tested the first hypothesis. Models 2 and 3, respectively, added the feelings of anxiety and depression scales and two-way interaction terms between feelings of anxiety and depression, and waves. There, we tested our second hypothesis, analyzing the influence of having had feelings of anxiety and depression on psychotropic drugs use. Models 4 and 5 are presented in Table 2. Model 4 assesses the effect of a possible educational gradient on psychotropic drugs use, which tested hypothesis 3. Model 5 then includes three-way interactions between wave, feelings of anxiety and depression, and educational level, building on the models that previously added two-way interaction terms between the other variables. We assessed the goodness of fit of our models via loglikelihood and the Hosmer-Lemeshow test.

**Table 1:** Multinomial logistic regression results for association between feelings of anxiety and depression and the use of psycholeptics (outcome 1), psychoanaleptics (outcome 2), and both psycholeptic and psychoanaleptic medication (outcome 3), in reference to use of neither psycholeptics nor psychoanaleptics. Own calculations via the Belgian Health Interview Survey, 2004–2013

	*Outcome* (1)*: use of psycholeptic medication (N05)*								
	*Model 1*			*Model 2*			*Model 3*		
*Multinomial logistic regression results*	*OR*	*Sig.*	*95% C.I.*	*OR*	*Sig.*	*95% C.I.*	*OR*	*Sig.*	*95% C.I.*
Intercept	0.015	***		0.002	***		0.001	***	
Waves (ref. 2008)									
2004	1.064		0.887, 1.276	1.103		0.897, 1.357	1.470		0.848, 2.550
2013	0.711	**	0.584, 0.866	0.571	***	0.451, 0.724	0.942		0.159, 1.710
Anxiety or depression feelings									
Anxiety				2.129	***	1.679, 2.701	2.070	**	1.358, 3.154
Depression				1.668	***	1.342, 2.073	2.033	***	1.399, 2.954
Wave (2008 = ref)*anxiety									
2004*anxiety							1.103		0.627, 1.943
2013*anxiety							1.019		0.557, 1.865
Wave(2008 = ref)*depression									
2004*depression							0.770		0.461, 1.286
2013*depression							0.728		0.418, 1.268
Social correlates									
Female gender (ref. male)	1.479	***	1.264, 1.727	1.258	*	1.047, 1.511	1.257	*	1.046, 1.510
Age	1.052	*	1.007, 1.100	1.059	*	1.006, 1.114	1.060	*	1.007, 1.116
Age^2^	1.000		0.999, 1.000	1.000		0.999, 1.000	1.000		0.999, 1.000
	*Outcome 2: use of psychoanaleptic medication (N06)*								
	*Model 1*			*Model 2*			*Model 3*		
*Multinomial logistic regression results*	*OR*	*Sig.*	*95% C.I.*	*OR*	*Sig.*	*95% C.I.*	*OR*	*Sig.*	*95% C.I.*
Intercept	0.007	***		0,001	***		0.001	***	
Waves (ref. 2008)									
2004	0.942		0.748, 1.187	0.950		0.738, 1.223	1.160		0.600, 2.243
2013	1.073		0.860, 1.338	0.434		0.703, 1.163	1.579		0.837, 2.980
Anxiety or depression feelings									
Anxiety				1.358	*	1.038, 1.777	1.227		0.757, 1.988
Depression				2.069	***	1.630, 2.626	2.682	***	1.778, 4.045
Wave (2008 = ref)*anxiety									
2004*anxiety							1.145		0.580, 2.259
2013*anxiety							0.122		0.635, 2.336
Wave(2008 = ref)*depression									
2004*depression							0.787		0.435, 1.426
2013*depression							0.605		0.341, 1.073
Social correlates									
Female gender (ref. male)	1.932	***	1.608, 2.322	1.522	***	1.235, 1.876	1.519	***	1.232, 1.872
Age	1.109	***	1.057, 1.164	1.135	***	1.073, 1.200	1.137	***	1.075, 1.202
Age^2^	0.999	***	0.998, 0.999	0.999	***	0.998, 0.999	0.999	***	0.998, 0.999
	*Outcome 3: use of both psycholeptic and psychoanaleptic (N05 + N06)*								
	*Model 1*			*Model 2*			*Model 3*		
*Multinomial logistic regression results*	*OR*	*Sig.*	*95% C.I.*	*OR*	*Sig.*	*95% C.I.*	*OR*	*Sig.*	*95% C.I.*
Intercept	0.0004	***		0.00002	***		0.00002	***	
Waves (ref. 2008)									
2004	0.984		0.781, 1.238	1.073		0.816, 1.412	0.874		0.437, 1.751
2013	0.747	*	0.588, 0.950	0.561	***	0.415, 0.758	0.743		0.351, 1.573
Anxiety or depression feelings									
Anxiety				1.958	***	1.503, 2.550	2.106	***	1.311, 3.385
Depression				2.577	***	2.021, 3.285	2.526	***	1.651, 3.867
Wave (2008 = ref)*anxiety									
2004*anxiety							0.950		0.501, 1.802
2013*anxiety							0.855		0.442, 1.656
Wave(2008 = ref)*depression									
2004*depression							1.135		0.633, 2.033
2013*depression							0.962		0.522, 1.774
Social correlates									
Female gender (ref. male)	2.835	***	2.336, 3.441	2.033	***	1.605, 2.576	2.028	***	1.600, 2.570
Age	1.201	***	1.132, 1.274	1.204	***	1.120, 1.295	1.206	***	1.121, 1.297
Age^2^	0.998	***	0.998, 0.999	0.999	***	0.998, 0.999	0.999	***	0.998, 0.999
**Nagelkerke R-squared**	0.057			0.192			0.194		
**Loglikelihood**	2888.431	***		7686.289	***		7676.496	***	

* *p* < 0.05 ** *p* < 0.01 *** *p* < 0.001; *n* = 7214

**Table 2:** Multinomial logistic regression results for association between feelings of anxiety and depression, education and wave and the use of psycholeptics (outcome 1), psychoanaleptics (outcome 2), and both psycholeptics and psychoanaleptics (outcome 3), in reference to use of neither psycholeptics nor psychoanaleptics. Own calculations via the Belgian Health Interview Survey, 2004–2013

	Outcome 1: use of psycholeptic medication (N05)					
	Model 4			*Model 5*		
*Multinomial logistic regression results*	OR	sig.	*95% C.I.*	*OR*	*Sig.*	*95% C.I.*
Intercept	0.003	***		0.003	***	
Waves (ref. 2008)						
2004	1.079		0.875, 1.331	1.340		0.428, 4.199
2013	0.591	***	0.465, 0.751	0.406		0.095, 1.736
Feelings of anxiety	2.124	***	1.671, 2.699	1.350		0.603, 3.019
Feelings of depression	1.597	***	1.283, 1.989	2.045		0.932, 4.489
Education (ref. no diploma)						
Secondary school diploma	0.998		0.795, 1.253	0.585		0.204, 1.680
Higher education diploma	0.593	***	0.450, 0.782	0.260	*	0.070, 0.970
Wave (ref. 2008)*feelings of anxiety						
2004*feelings of anxiety				4.327	**	1.447, 12.936
2013*feelings of anxiety				1.467		0.408, 5.276
Wave (ref. 2008)*feelings of depression						
2004*feelings of depression				0.241	**	0.083, 0.698
2013*feelings of depression				0.813		0.227, 2.911
Wave (ref. 2008)* education (ref. no diploma)						
2004*secondary education diploma				0.709		0.355, 5.607
2013*secondary education diploma				3.932		0.049, 1.325
2004*higher education diploma				2.635		0.069, 2.075
2013*higher education diploma				1.662		0.082, 4.416
Education (ref. no diploma)* feelings of anxiety						
Secondary education diploma* feelings of anxiety				2.693	*	0.995, 7.289
Higher education diploma*feelings of anxiety				0.611		0.169, 2.208
Education (ref. no diploma)*feelings of depression						
Secondary education diploma* feelings of depression				0.614		0.242, 1.560
Higher education diploma*feelings of depression				2.658		0.811, 8.714
Wave (ref. 2008)* education (ref. no diploma)*feelings of anxiety						
2004*secondary education diploma*feelings of anxiety				0.111	**	0.029, 0.429
2013*secondary education diploma*feelings anxiety				0.338		0.074, 1.555
2004*higher education diploma*feelings of anxiety				0.339		0.056, 2.073
2013*higher education diploma*feelings of anxiety				2.732		0.411, 18.149
Wave (ref. 2008)*education (ref. no diploma)* feelings of depression						
2004*secondary education diploma*feelings of depression				8.017	**	2.256, 28.489
2013*secondary education diploma*feelings of depression				1.343		0.307, 5.868
2004*higher education diploma*feelings of depression				1.615		0.305, 8.539
2013*higher education diploma*feelings of depression				0.294		0.048, 1.800
Gender (ref. male)	1.255	***	1.042, 1.512	1.257	*	1.042, 1.517
Age	1.055	*	1.001, 1.111	1.061	*	1.007, 1.118
Age^2^	1.000		1.000, 0.999	1.000		0.999, 1.000
	Outcome: use of psychoanaleptic medication (N06)					
	*Model 4*			*Model 5*		
*Multinomial logistic regression results*	*OR*	*Sig.*	*95% C.I.*	*OR*	*Sig.*	*95% C.I.*
Intercept	0.002	***		0.001	***	
Waves (ref. 2008)						
2004	1.001		0.773, 1.298	1.004		0.201, 5.027
2013	0.974		0.752, 1.261	1.422		0.227, 8.904
Feelings of anxiety	1.372	*	1.044, 1.804	1167		0.196, 2.137
Feelings of depression	2.037	***	1.599, 2.596	2.889		0.995, 7.742
Education (ref. no diploma)						
Secondary school diploma	0.946		0.700, 1.278	0.451		0.106, 1.917
Higher education diploma	1.002		0.726, 1.383	0.226		0.046, 1.119
Wave (ref. 2008)*feelings of anxiety						
2004*feelings of anxiety				1.828		0.380, 8.800
2013*feelings of anxiety				1.033		0.181, 5.890
Wave (ref. 2008)*feelings of depression						
2004*feelings of depression				0.665		0.176, 2.519
2013*feelings of depression				0.785		0.169, 3.636
Wave (ref. 2008)* education (ref. no diploma)						
2004*secondary education diploma				0.784		0.194, 8.391
2013*secondary education diploma				1.324		0.098, 5.802
2004*higher education diploma				2.899		0.045, 2.637
2013*higher education diploma				1.347		0.083, 6.662
Education (ref. no diploma)* feelings of anxiety						
Secondary education diploma* feelings of anxiety				2.828		0.713, 11.217
Higher education diploma*feelings of anxiety				1.274		0.275, 5.914
Education (ref. no diploma)*feelings of depression						
Secondary education diploma* feelings of depression				0.701		0.218, 2.252
Higher education diploma*feelings of depression				1.948		0.502, 7.565
Wave (ref. 2008)* education (ref. no diploma)*feelings of anxiety						
2004*secondary education diploma*feelings of anxiety				0.540		0.085, 3.427
2013*secondary education diploma*feelings anxiety				0.918		0.128, 6.611
2004*higher education diploma*feelings of anxiety				0.799		0.099, 6.456
2013*higher education diploma*feelings of anxiety				2.109		0.253, 17.571
Wave (ref. 2008)*education (ref. no diploma)* feelings of depression						
2004*secondary education diploma*feelings of depression				1.496		0.308, 7.274
2013*secondary education diploma*feelings of depression				0.859		0.152, 4.862
2004*higher education diploma*feelings of depression				0.639		0.103, 3.976
2013*higher education diploma*feelings of depression				0.455		0.069, 2.996
Gender (ref. male)	1.508	***	1.219, 1.865	1.518	***	1.225, 1.880
Age	1.138	***	1.074, 1.206	1.143	***	1.078, 1.212
Age^2^	0.999	***	0.998, 0.999	0.999	***	0.998, 0.999
	Outcome 3: use of both psycholeptic and psychoanaleptic medication (N05 + N06)					
	*Model 4*			*Model 5*		
*Multinomial logistic regression results*	*OR*	*sig.*	*95% C.I.*	*OR*	*Sig.*	*95% C.I.*
Intercept	0.00002	***		0.00002	***	
Waves (ref. 2008)						
2004	1.043		0.789, 1.377	1.595		0.319, 7.974
2013	0.567	***	0.418, 0.770	1.518		0.254, 9.080
Feelings of anxiety	1.918	***	1.467, 2.509	1.314		0.492, 3.507
Feelings of depression	2.532	***	1.980, 3.238	2.816	*	1.065, 7.444
Education (ref. no diploma)						
Secondary school diploma	1.072		0.790, 1.454	0.910		0.207, 3.996
Higher education diploma	0.714		0.495, 1.030	0.327		0.059, 1.815
Wave (ref. 2008)*feelings of anxiety						
2004*feelings of anxiety				1.457		0.380, 5.579
2013*feelings of anxiety				1.063		0.262, 4.311
Wave (ref. 2008)*feelings of depression						
2004*feelings of depression				0.650		0.185, 2.290
2013*feelings of depression				0.803		0.200, 3.229
Wave (ref. 2008)* education (ref. no diploma)						
2004*secondary education diploma				0.307		0.495, 21.361
2013*secondary education diploma				0.563		0.229, 13.782
2004*higher education diploma				1.250		0.090, 7.070
2013*higher education diploma				0.454		0.188, 25.711
Education (ref. no diploma)* feelings of anxiety						
Secondary education diploma* feelings of anxiety				3.067		0.932, 10.086
Higher education diploma*feelings of anxiety				0.621		0.154, 2.502
Education (ref. no diploma)*feelings of depression						
Secondary education diploma* feelings of depression				0.496		0.160, 1.540
Higher education diploma*feelings of depression				3.158		0.830, 12.009
Wave (ref. 2008)* education (ref. no diploma)*feelings of anxiety						
2004*secondary education diploma*feelings of anxiety				0.379		0.076, 1.899,
2013*secondary education diploma*feelings anxiety				0.508		0.094, 2.741
2004*higher education diploma*feelings of anxiety				1.298		0.174, 9.694
2013*higher education diploma*feelings of anxiety				1.347		0.191, 9.485
Wave (ref. 2008)*education (ref. no diploma)* feelings of depression						
2004*secondary education diploma*feelings of depression				3.709		0.836, 16.455
2013*secondary education diploma*feelings of depression				1.637		0.319, 8.404
2004*higher education diploma*feelings of depression				0.534		0.082, 3.470
2013*higher education diploma*feelings of depression				0.604		0.096, 3.799
Gender (ref. male)	2.094	***	1.647, 2.663	2.130	***	1.671, 2.716
Age	1.213	***	1.125, 1.308	1.225	***	1.135, 1.322
Age^2^	0.998	***	0.998, 0.999	0.998	***	0.998, 0.999
**Nagelkerke R-squared**	0.196			0.211		
**Loglikelihood**	981.285	***		1063.395	***	

* *p* < 0.05 ** *p* < 0.01 *** *p* < 0.001; *n* = 7214

Finally, a number of sensitivity analyses were performed. First, time trends in psycholeptics and psychoanaleptics use for respondents with AD and DD were also estimated, by categorizing respondents as having AD or DD when the scale total was more than 2 on the respective anxiety and depression scales, as is suggested by HIS [49]. Second, help-seeking behavior was added to the models, in order to examine whether and how this variable mediates the established associations. Results are presented in the Appendix (Fig. A1 and Table A2) and discussed in the text. All analyses were performed with the IBM SPSS Statistics software package (version 27).

## Results

### Descriptives

As Fig. 1 shows, the use of psycholeptic drugs has decreased when comparing the three waves, while the use of psychoanaleptic drugs has increased modestly. Moreover, both feelings of anxiety and depression have been on the rise. Herein, feelings of depression tended to rise more substantially compared to feelings of anxiety. Fig. A1 additionally shows that, while the likelihood of psycholeptics use decreased over time, psychoanaleptics use increased substantially for persons with AD. In 2013, persons with AD consumed more psychoanaleptics than psycholeptics. The use of a combination of both psycholeptic and psychoanaleptic drugs shows a general downward trend.

Figure 2 shows that persons with a lower level of education tended to be more likely to consume either or both psycholeptic and psychoanaleptic drugs. Therein, persons with no education systematically tended to be the most likely to consume psycholeptic drugs. Moreover, persons with the highest level of education were least likely to consume either type of psychotropic drugs, throughout the observed period. The consumption of a combination of psycholeptics and psychoanaleptics shows a general downward trend in the observed period, though not for persons with no diploma.

### Multinomial logistic regression results

Multinomial regression analysis pointed to a significant decrease in likelihood for psycholeptics (OR = 0.711, 95% CI [0.584, 0.866]) or a combination of psycholeptics and psychoanaleptics (OR = 0.747, 95% CI [0.588, 0.950]) consumption for 2013 when comparing to the reference year 2008, (Model1, Table 1). Persons were also more likely to consume psycholeptics (OR = 1.064, 95% CI [0.887, 1.276]) in 2004. Adding feelings of anxiety and depression to the analyses (Model2) further widened the gap between waves, for both the psycholeptic as well as the combination outcome. Furthermore, having more feelings of anxiety increased the odds (OR = 2.129, 95% CI [1.679, 2.701]) of consuming psycholeptic drugs compared to having consumed no psychotropic drugs, while this effect was weaker for feelings of depression (OR = 1.668, 95% CI [1.342, 2.073]). This effect is reversed for the psychoanaleptic outcome, though with a wider gap between feelings of anxiety (OR = 1.358, 95% CI [1.038, 1.777]) and feelings of depression (OR = 2.069, 95% CI [1.630, 2.626]). Having more feelings of depression increased the odds of taking a combination of both psycholeptic and psychoanaleptic drugs, compared to having more feelings of anxiety. Model 3 added interaction terms between wave and feelings of anxiety and wave and feelings of depression. Though, none of the interaction terms were significant. Having had a higher education (Model 4) did lead to a decrease in odds for consuming psycholeptic drugs (OR = 0.593, 95% CI [0.450, 0.782]). This effect was reversed for psychoanaleptic drugs use, though it was not significant. Model 5 shows that having more feelings of anxiety in 2004, led to a significant increase in the odds of psycholeptic use (OR = 4.327, 95% CI [1.447, 12.936]). Having more feelings of depression in 2004, however, led to a significant decrease in the odds (OR = 0.241, 95% CI [0.083, 0.698]). Moreover, persons with a secondary level diploma and more feelings of anxiety had greater odds (OR = 2.693, 95% CI [0.995, 7.289]) to consume psycholeptic drugs as well. The odds increased significantly for persons having more feelings of depression and a secondary diploma in 2004 (OR = 8.017, 95% CI [2.256, 28.489]), compared to having feelings of anxiety (OR = 0.111, 95% CI [0.029, 0.429]).

Lastly, an additional sensitivity analysis (See Appendix Table A2) revealed that, when compared to model 4, the effect of having more feelings of anxiety and depression slightly decreased when compared to the help-seeking reference category, which is having visited neither GP nor psychologist in the past year, suggesting a mediating effect. The effect of having visited both a GP and psychologist increased the odds of taking psycholeptic drugs (OR = 3.417, 95% CI [2.137, 5.464]), while having visited only a psychologist had a similar effect (OR = 3.323, 95% CI [1.033, 10.689]). However, having visited only a GP had no significant effect on whether respondents used psycholeptics, compared to having used neither psycholeptics or psychoanaleptics. For psychoanaleptic use, effects were more outspoken. Having visited only a psychologist had the biggest effect, increasing odds 16-fold, while having visited both a psychologist and GP also increased odds tenfold. An important caveat, however, is that the sample size for having visited only a psychologist is rather small (*n* = 83) when compared to the other categories. Having visited only a GP had a significant effect (OR = 2.609, 95% CI [1.477, 4.608]), too, suggesting GP’s were more likely to prescribe psychoanaleptics than psycholeptics. For the combination outcome, having visited both GP and psychologist had a significant effect, increasing the odds by a factor of 8.6.

## Discussion

Distinguishable patterns of psychotropic drugs use for mitigating feelings of anxiety and depression have been described throughout the observed period. While both feelings of anxiety and depression were on the rise in Belgium, psycholeptics became more obscured, while psychoanaleptics have been booming. We can therefore confirm our first hypothesis. This trend was also confirmed by other researchers in other contexts, who noted a shift to psychoanaleptic drugs (specifically antidepressants) occurred in the late 2000s [27, 50].

The consumption of psycholeptics and psychoanaleptics was influenced not only by scientific developments, but also by how society perceives these types of treatments [51]. This could be ascribed to three reasons. First, psycholeptic drugs (such as benzodiazepines) are notoriously addictive [52]. It is argued that the prolonged use of this type of medication ultimately leads to a dependency, fueled by either long-term, ill-managed treatment by, e.g., general practitioners, which could result in addiction or the use of illicit drugs [53, 54]. This rhetoric is often bloated by the media (see e.g. [55]), further fueling fears and doubt surrounding this treatment method. Second, the use of psycholeptic drugs is associated with substantial health problems, such as cognitive decline, especially among the elderly [56], and withdrawal syndrome [52]. Therefore, short-term psycholeptic use is most often advised. Third, developments in psychoanaleptic drugs treatment systematically showed promising results in the last 30 years [57]. However, while the first antidepressants, for instance, were received with great enthusiasm, their side-effects were often overlooked and minimized when compared to psycholeptics [25].

Others (e.g. [25, 27, 58]) argue that the use of benzodiazepines (and other psycholeptic drugs) should be reassessed, claiming its use could lead to benefits that outweigh possibilities of addiction and other health problems. The decrease in psycholeptic drugs use and its prescribing is, at least partially, the result of (societal) biases towards it. In addition, the pharmaceutical industry has increasingly favored, for instance, antidepressants over benzodiazepines in the last two decades [24]. This has led to rigorous new treatment protocols and even legislation to help restrict psycholeptic drugs use. Shifts in treatment are also caused by the systematic reframing of both disorders [5, 14, 22]. For instance, the reframing of DD, particularly by the APA’s DSM-III, changed the perception on mental disorders drastically [16]. DD became an umbrella term for a large number of (mood) disorders, causing prevalence rates to increase substantially [5]. All the while, AD was being subdivided into multiple new disorders [13]. In addition, the APA argued that treatment with psychoanaleptics was more suited to these new (anxiety) diagnoses [23, 25]. This study shows that it could have rather been the other way around.

Our research additionally established the existence of a social gradient within the consumption of psycholeptic and psychoanaleptic drugs, confirming our third hypothesis. Higher educated persons consumed less of either (or both) psycholeptic and psychoanaleptic drugs throughout all of the observed period. This could firstly be explained by the already existing social gradient in the distribution of mental illnesses, with persons having enjoyed less education being more susceptible to them in the first place [59, 60]. Nielsen, Hansen [59] argue that medication consumption is congruent with this distribution, resulting in more consumption with those that are lower educated compared to those who are higher educated. Secondly, persons with less education often have more difficulty navigating health care systems, leading them to accept treatment methods that are most easily available, usually via ambulatory care [8, 61]. In the United States, for example, acute anxiety attacks are often relieved with a one-time psycholeptics prescription [62]. Though, this practice is becoming less common for persons with lower SES due to (sometimes unfounded) suspicions of substance abuse [54]. Thirdly, the prescribing behavior of clinicians and subsequent treatment of mental disorders varies between patients with different educational backgrounds [63]. This could be due to stigma revolving around psychiatric treatment [51, 60], which was already discussed specifically in the case of psycholeptics and benzodiazepines, but is also true for most medication-based treatments [25, 37, 63].

Moreover, the use of psychoanaleptics is most prevalent in persons with the highest educational level. Newer types of treatment, such as psychoanaleptics, are often more readily available for persons with a higher educational level (e.g. [64]), while they are deemed less hazardous and thus less stigmatizing [25]. Our results suggest that the shift to a higher likelihood of psychoanaleptic drugs consumption in persons with the highest education already happened before the observed period, but that this shift is now occurring for persons with secondary education, describing a diffusion of psychotropic innovations. Persons with higher education, furthermore, increasingly opt for alternative types of treatment to prevent stigma, while taking on an active patient role, while persons with lower education generally still take on a more passive patient role [65].

Finally, the literature suggests there are recent trends of demedicalization in the treatment (or prevention) of mental illness [9, 44]. We performed a sensitivity analysis to investigate differences in the medication use outcome when controlled for different forms of help-seeking behavior. Therein, having visited only a GP returned the lowest likelihood of psycholeptic or psychoanaleptic drugs consumption, suggesting primary care prescriptions for psycholeptics or psychoanaleptics are less common than when patients also visited a psychologist. This could indicate that medicinal treatments are increasingly combined with talking therapy, as is most often advised nowadays [23, 57, 66]. However, since we could not include a variable for psychiatry or alternative medicine, evidence to support these trends is lacking in comprehensiveness.

While interpreting the results, some limitations of this study are worth noting. First, the SCL-90-R measurement could be partially mediated by medication use, leading to response-biases. Second, the observed period is rather limited in duration. Third, this study did not take into account the high comorbidity between AD and DD. Fourth, our ideal-typical distinction between psycholeptic and psychoanaleptic drugs excludes other medication types or combinations of psychotropic drugs that are often used to treat both disorders. This study could however be used as a starting point to prompt further research, focusing on different medication subgroups (or perhaps even brands). Fifth, as our analyses consist of some higher-order interactions, some variables in our equation have a low cell-count, leading to higher standard errors and wide confidence intervals. Finally, as the Belgian Health Interview Survey did not offer consistently measured variables for having visited a psychiatrist or alternative practitioner our research thereof is limited. We therefore suggest further research, following up on more recent trends of demedicalization, via e.g., more elaborate mediation analyses.

## Conclusions

In conclusion, this study provides evidence for a shift in the medicalization and pharmaceuticalizaiton of both feelings of anxiety and depression, and that the medicalization of these feelings was dissimilar, while pharmaceuticalization was indeed converging towards another. Using the ideal-typical distinction of psycholeptics and psychoanaleptics allowed us to test the medicalization framework as a means to unambiguously assess differences between both disorders. Finally, we established a social gradient that partially mediated these shifts. This, by itself, means that treatment for feelings of anxiety and depression was perceived differently for different persons (in this case depending on educational level).

## Supplementary Information

**Additional file 1: Table A1.** Descriptives (unstandardized, weighted data). Own calculations via the Belgian Health Interview Survey, 2004-2013. **Table A2.** Multinomial logistic regression results for association between mental health care use and the use of psycholeptics, psychoanaleptics and both psycholeptics and psychoanaleptics, in reference to use of neither psycholeptics nor psychoanaleptics. Own calculations via the Belgian Health Interview Survey, 2004-2013. **Figure A1.** Proportion of respondents with DD and AD (measured via a cut-off of higher than 2-points on the SCL-90-R scale, consuming medicines. Own calculations via the Belgian Health Interview Survey, 2004-2013.

## Data Availability

Data was formally obtained via BHIS, the Belgian Health Interview Survey, as collected by Sciensano.

## References

[1] Kessler RC, Aneshensel CS, Phelan JC, Bierman A (2013). Overview of descriptive epidemiology of mental disorders. Handbook of the sociology of mental health.

[2] Craske MG, Stein MB (2016). Anxiety. Lancet.

[3] Kessler RC, Petukhova M, Sampson NA, Zaslavsky AM, Wittchen H-U (2012). Twelve-month and lifetime prevalence and lifetime morbid risk of anxiety and mood disorders in the United States. Int J Methods Psychiatr Res.

[4] Gisle L, Drieskens S, Demarest S, Van der Heyden J (2018). Geestelijke gezondheid: gezondheidsenquête 2018.

[5] Horwitz AV (2010). How an age of anxiety became an age of depression. Milbank Q.

[6] Mwinyi J, Pisanu C, Castelao E, Stringhini S, Preisig M, Schiöth HB (2017). Anxiety disorders are associated with low socioeconomic status in women but not in men. Womens Health Issues.

[7] Link BG, Phelan J. Social conditions as fundamental causes of disease. J Health Soc Behav. 1995;35:80–94. 7560851

[8] Pampel FC, Krueger PM, Denney JT (2010). Socioeconomic disparities in health behaviors. Annu Rev Sociol.

[9] Clarke AE, Shim JK, Mamo L, Fosket JR, Fishman JR (2003). Biomedicalization: Technoscientific transformations of health, illness, and U.S. Biomed Am Sociol Rev.

[10] Horwitz AV, Wakefield J. All we have to fear: Psychiatry’s transformation of natural anxieties into mental disorders. Oxford: Oxford University Press; 2012.

[11] Horwitz AV, Wakefield J (2007). The loss of sadness: how psychiatry transformed normal sorrow into depressive disorder.

[12] Bruce ML, Raue PJ, Aneshensel CS, Phelan JC, Bierman A (2013). Mental Illness as psychiatric disorder. Handbook of the sociology of mental health.

[13] Starkstein S (2018). A conceptual and therapeutic analysis of fear.

[14] Conrad P (2005). The shifting Engines of Medicalization. J Health Soc Behav.

[15] Hirschfeld RM (2001). The comorbidity of major depression and anxiety disorders: recognition and Management in Primary Care. Prim Care Companion J Clin Psychiatry.

[16] Horwitz AV (2007). Transforming normality into pathology: the DSM and the outcomes of stressful social arrangements. J Health Soc Behav.

[17] Conrad P, Barker KK (2010). The social construction of Illness: key insights and policy implications. J Health Soc Behav.

[18] Schnittker J, Aneshensel CS, Phelan JC, Bierman A (2013). Public Beliefs About Mental Illness. Handbook of the sociology of mental health.

[19] Deacon BJ (2013). The biomedical model of mental disorder: a critical analysis of its validity, utility, and effects on psychotherapy research. Clin Psychol Rev.

[20] Scott S (2006). The medicalisation of shyness: from social misfits to social fitness. Sociol Health Illn.

[21] Pies RW (2014). The bereavement exclusion and DSM-5: an update and commentary. Innov Clin Neurosci.

[22] Horwitz AV (2011). Creating an age of depression: the social construction and consequences of the major depression diagnosis. Soc Mental Health.

[23] Bandelow B, Michaelis S, Wedekind D (2017). Treatment of anxiety disorders. Dialogues Clin Neurosci.

[24] Spence D (2016). Bad medicine: the rise and rise of antidepressants. Br J Gen Pract.

[25] Balon R, Starcevic V, Silberman E, Cosci F, Dubovsky S, Fava GA (2020). The rise and fall and rise of benzodiazepines: a return of the stigmatized and repressed. Braz J Psychiatry.

[26] Cohen D, McCubbin M, Collin J, Perodeau G (2001). Medications as social phenomena. Health.

[27] Offidani E, Guidi J, Tomba E, Fava GA (2013). Efficacy and tolerability of benzodiazepines versus antidepressants in anxiety disorders: a systematic review and meta-analysis. Psychother Psychosom.

[28] Ehrenberg A (2010). The weariness of the self: diagnosing the history of depression in the contemporary age.

[29] Declercq T, Habraken H, Van Den Ameele H, Callens J, De Lepeleire J, Cloetens H (2016). Depressie Bij Volwassenen: Herziening in opdracht van de commissie richtlijnen.

[30] Kurko T, Saastamoinen LK, Tuulio-Henriksson A, Taiminen T, Tiihonen J, Airaksinen M (2018). Trends in the long-term use of benzodiazepine anxiolytics and hypnotics: a national register study for 2006 to 2014. Pharmacoepidemiol Drug Saf.

[31] de Looper M, Lafortune G (2009). Measuring disparities in health status and in access and use of health care in OECD Countries.

[32] De Cock J, RIZIV (2014). Belgische verzekering voor geneeskundige verzorging en uitkeringen: Mijlpalen van het verleden, bakens voor de toekomst.

[33] Davies K, Stremikis K, Squires D, Schoen C. Mirror, Mirror on the wall: how the performance of the U.S. Health Care System Compares Internationally. Oxford The Commonwealth Fund; 2014.

[34] Field MG, Powell FD, Wessen AF (1999). Comparative health systems and the convergence hypothesis: the dialectics of universalism and particularism. Health Care Systems in Transition: an international perspective.

[35] BelPEP (2015). Globale visienota en actieplan van de 3 werkgroepen.

[36] Bell AV (2016). The margins of medicalization: diversity and context through the case of infertility. Soc Sci Med.

[37] Sleath B, Tina Shih Y-C (2003). Sociological influences on antidepressant prescribing. Soc Sci Med.

[38] Muntaner C, Ng E, Vanroelen C, Christ S, Eaton WW, Aneshensel CS, Phelan JC, Bierman A (2013). Social stratification, social closure, and social class as determinants of mental health disparities. Handbook of the sociology of mental health.

[39] McLeod JD, Aneshensel CS, Phelan JC, Bierman A (2013). Social stratification and inequality. Handbook of the sociology of mental health.

[40] Abraham J (2010). Pharmaceuticalization of society in context: theoretical, empirical and health dimensions. Sociology.

[41] Bjelland I, Krokstad S, Mykletun A, Dahl A, Tell G, Tambs K (1982). Does higher education protect against anxiety and depression? The HUNT study. Soc Sci Med.

[42] Dudal P, Bracke P. Absolute and relative educational inequalities in depression in Europe. Int J Public Health. 2016;61:787–95. 10.1007/s00038-016-0837-5 27220547

[43] Demyttenaere K, Bonnewyn A, Bruffaerts R, De Girolamo G, Gasquet I, Kovess V (2008). Clinical factors influencing the prescription of antidepressants and benzodiazepines: results from the European study of the epidemiology of mental disorders (ESEMeD). J Affect Disord.

[44] Hofmann SG, Sawyer AT, Witt AA, Oh D (2010). The effect of mindfulness-based therapy on anxiety and depression: a meta-analytic review. J Consult Clin Psychol.

[45] Demarest S, Van der Heyden J, Charafeddine R, Drieskens S, Gisle L, Tafforeau J (2013). Methodological basics and evolution of the Belgian health interview survey 1997–2008. Arch Public Health.

[46] World Health Organization (2018). Structure and principles.

[47] Derogatis LR (1994). SCL-90-R : symptom checklist-90-R : administration, scoring & procedures manual.

[48] Mirowsky J, Ross CE (1992). Age and depression. J Health Soc Behav.

[49] Gisle L, Van der Heyden J, Charafeddine R (2014). Geestelijke gezondheid. Gezondheidsenquête 2013.

[50] John U, Sebastian E, Völzke H, Grabe H, Freyberger H, Alte D (2008). Estimation of psycholeptic and psychoanaleptic medicine use in an adult general population sample using the anatomical therapeutic chemical classification. Int J Methods Psychiatr Res.

[51] Angermeyer MC, Breier P, Dietrich S, Kenzine D, Matschinger H (2005). Public attitudes toward psychiatric treatment. Soc Psychiatry Psychiatr Epidemiol.

[52] Brett J, Murnion B (2015). Management of benzodiazepine misuse and dependence. Aust Prescr.

[53] Ashton H (2005). The diagnosis and management of benzodiazepine dependence. Curr Opin Psychiatry.

[54] Schmitz A (2016). Benzodiazepine use, misuse, and abuse: A review. Mental Health Clin.

[55] Garrison A (2018). Antianxiety drugs — often more deadly than opioids — are fueling the next drug crisis in US.

[56] Paterniti S, Dufouil C, Alpérovitch A. Long-term benzodiazepine use and cognitive decline in the elderly: the epidemiology of vascular aging study. J Clin Psychopharmacol. 2002;22(3):285–93. 10.1097/00004714-200206000-00009 12006899

[57] Cuijpers P, Stringaris A, Wolpert M (2020). Treatment outcomes for depression: challenges and opportunities. Lancet Psychiatry.

[58] Silberman E, Balon R, Starcevic V, Shader R, Cosci F, Fava GA (2021). Benzodiazepines: it's time to return to the evidence. Br J Psychiatry.

[59] Nielsen MW, Hansen EH, Rasmussen NK (2004). Patterns of psychotropic medicine use and related diseases across educational groups: national cross-sectional survey. Eur J Clin Pharmacol.

[60] Seifert J, Führmann F, Reinhard MA, Engel RR, Bernegger X, Bleich S, et al. Sex differences in pharmacological treatment of major depressive disorder: results from the AMSP pharmacovigilance program from 2001 to 2017. J Neural Transm. 2021;128(6):827–43. 10.1007/s00702-021-02349-5 PMC8205885 33977402

[61] Kangovi S, Barg FK, Carter T, Long JA, Shannon R, Grande D (2013). Understanding why patients of low socioeconomic status prefer hospitals over ambulatory care. Health Aff.

[62] Agarwal SD, Landon BE (2019). Patterns in outpatient benzodiazepine prescribing in the United States. JAMA Netw Open.

[63] Dorner TE, Mittendorfer-Rutz E (2017). Socioeconomic inequalities in treatment of individuals with common mental disorders regarding subsequent development of mental illness. Soc Psychiatry Psychiatr Epidemiol.

[64] Drake R, Skinner J, Goldman HH (2008). What explains the diffusion of treatments for mental illness?. Am J Psychiatry.

[65] Smith SG, Pandit A, Rush SR, Wolf MS, Simon CJ (2016). The role of patient activation in preferences for shared decision making: results from a National Survey of U.S. Adults J Health Commun.

[66] Bleakley S, Davies SJC (2014). The pharmacological management of anxiety disorders. Prog Neurol Psychiatry.

