# Supplementary material for: An examination of the medicalization and pharmaceuticalization processes of anxiety and depressive disorders in Belgium between 2004 and 2013: how may both disorders be intertwined?
Source: Arch Public Health. 2022 Aug 15;80:191. doi: 10.1186/s13690-022-00943-x (PMC9377052; doi:10.1186/s13690-022-00943-x)

**APPENDIX**

**Table A1:** Descriptives (unstandardized, weighted data). Own calculations via the Belgian Health Interview Survey, 2004-2013.

|  | | **N** | **%** | **Mean** | **S.D.** | **χ² ^a^** |
| --- | --- | --- | --- | --- | --- | --- |
| Use of medicines in the past 24 hours | | 7,214 |  |  |  | *** |
|  | No psycholeptics or psychoanaleptics (ref) | 543 | 75.270% |  |  |  |
|  | Only psycholeptics (N05) | 784 | 10.868% |  |  |  |
|  | Only psychoanaleptics (N06) | 525 | 7.278% |  |  |  |
|  | Both psycholeptics and psychoanaleptics (N05+N06) | 475 | 6.584% |  |  |  |
| Wave | | 13,575 |  |  |  | - |
|  | 2008 (ref) | 4,217 | 31.064% |  |  |  |
|  | 2004 | 5,006 | 36.877% |  |  |  |
|  | 2013 | 4,352 | 32.059% |  |  |  |
| Anxiety feelings SCL-90-R | | 11,037 |  | 1.296 | 0.519 | *** |
| Depressive feelings SCL-90-R | | 11,056 |  | 1.407 | 0.595 | *** |
| Level of education | | 13,005 |  |  |  | *** |
|  | No diploma (ref) | 2,072 | 15.932% |  |  |  |
|  | Secondary education diploma | 6,492 | 49.919% |  |  |  |
|  | High education diploma | 4,441 | 34.148% |  |  |  |
| Help-seeking behavior in the past 12 months | | 13,575 |  |  |  | *** |
|  | Visited no GP or psychologist (ref) | 2,744 | 20.865% |  |  |  |
|  | Visited only a GP | 9,703 | 73.781% |  |  |  |
|  | Visited both a GP and a psychologist | 704 | 5.186% |  |  |  |
| Age | | 13,575 |  | 48.942 |  | *** |
| Gender | | 13,575 |  |  |  | *** |
|  | Male (ref) | 4,450 | 32.781% |  |  |  |
|  | Female | 9,125 | 67.219% |  |  |  |

^a^ Pearson’s chi-square was calculated for the association between each variable and wave.
* p<0.05 ** p<0.01 *** p<0.001

**Table A2.** Multinomial logistic regression results for association between mental health care use and the use of psycholeptics, psychoanaleptics and both psycholeptics and psychoanaleptics, in reference to use of neither psycholeptics nor psychoanaleptics. Own calculations via the Belgian Health Interview Survey, 2004-2013.

|  |  | *Use of psycholeptics* | |  | *Use of psychoanaleptics* | |  | *Use of both psycholeptic and psychoanaleptics* | |
| --- | --- | --- | --- | --- | --- | --- | --- | --- | --- |
|  |  |  |  |  |  |  |  |  |  |
|  | | *OR* | *Sig.* |  | *OR* | *Sig.* |  | *OR* | *Sig.* |
| Intercept | | 0.002 | *** |  | 0.0003 | *** |  | 0.000 | *** |
| Waves (ref. 2008) | |  |  |  |  |  |  |  |  |
|  | 2004 | 1.105 |  |  | 1.037 |  |  | 1.108 |  |
|  | 2013 | 0.578 | *** |  | 0.929 |  |  | 0.549 | *** |
| Anxiety or depression feelings | |  |  |  |  |  |  |  |  |
|  | Anxiety (max. 5) | 2.091 | *** |  | 1.348 | * |  | 1.905 | *** |
|  | Depression (max. 5) | 1.480 | ** |  | 1.811 | *** |  | 2.253 | *** |
| Highest individual diploma | |  |  |  |  |  |  |  |  |
| (ref. no diploma) | |  |  |  |  |  |  |  |  |
|  | Secondary school diploma | 0.991 |  |  | 0.922 |  |  | 1.042 | * |
|  | Higher education diploma | 0.568 | *** |  | 0.925 |  |  | 0.648 |  |
| Help-seeking last 12 months (ref. neither) | |  |  |  |  |  |  |  |  |
|  | Only GP | 1.026 |  |  | 2.609 | ** |  | 1.707 |  |
|  | Only psychologist | 3.323 | * |  | 16.414 | *** |  | 4.345 |  |
|  | Both GP and psychologist | 3.417 | *** |  | 10.195 | *** |  | 8.592 | *** |
| Social correlates | |  |  |  |  |  |  |  |  |
|  | Female gender (ref. male) | 1.248 | *** |  | 1.477 | *** |  | 2.081 | *** |
|  | Age | 1.065 | *** |  | 1.146 | *** |  | 1.227 | *** |
|  | Age² | 1.000 |  |  | 0.999 | *** |  | 0.998 | *** |

* p<0.05 ** p<0.01 *** p<0.001; n=7214.

**Figure A1** Proportion of respondents with DD and AD (measured via a cut-off of higher than 2-points on the SCL-90-R scale, consuming medicines. Own calculations via the Belgian Health Interview Survey, 2004-2013.


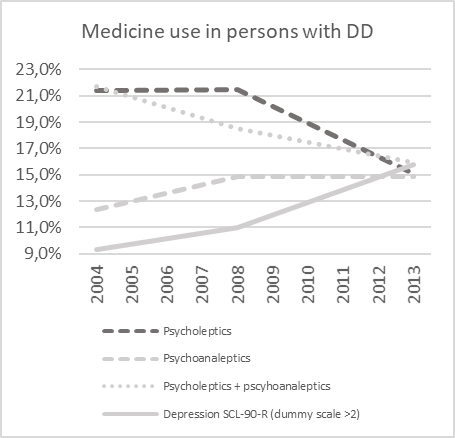

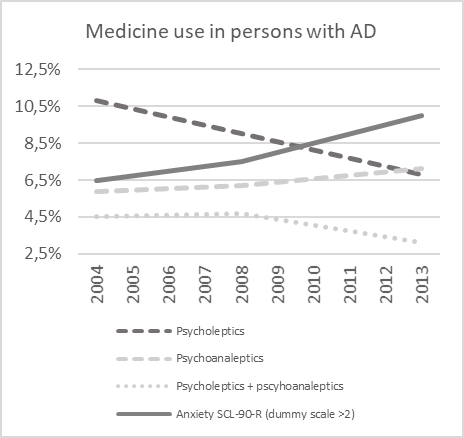

Supplement: Supplementary file 1 — Additional file 1: Table A1. Descriptives (unstandardized, weighted data). Own calculations via the Belgian Health Interview Survey, 2004-2013. Table A2. Multinomial logistic regression results for association between mental health care use and the use of psycholeptics, psychoanaleptics and both psycholeptics and psychoanaleptics, in reference to use of neither psycholeptics nor psychoanaleptics. Own calculations via the Belgian Health Interview Survey, 2004-2013. Figure A1. Proportion of respondents with DD and AD (measured via a cut-off of higher than 2-points on the SCL-90-R scale, consuming medicines. Own calculations via the Belgian Health Interview Survey, 2004-2013. [file 13690_2022_943_MOESM1_ESM.docx]
